# Novel Alleles for Combined Drought and Heat Stress Tolerance in Wheat

**DOI:** 10.3389/fpls.2019.01800

**Published:** 2020-01-31

**Authors:** Jessica Schmidt, Penny J. Tricker, Paul Eckermann, Priyanka Kalambettu, Melissa Garcia, Delphine Fleury

**Affiliations:** School of Agriculture, Food and Wine, The University of Adelaide, Adelaide, SA, Australia

**Keywords:** genome-wide association, quantitative trait loci, near-isogenic lines, *Triticum*, abiotic, genetic diversity

## Abstract

Drought and heat waves commonly co-occur in many wheat-growing regions causing significant crop losses. The identification of stress associated quantitative trait loci, particularly those for yield, is problematic due to their association with plant phenology and the high genetic × environment interaction. Here we studied a panel of 315 diverse, spring type accessions of bread wheat (*Triticum aestivum*) in pots in a semi-controlled environment under combined drought and heat stress over 2 years. Importantly, we treated individual plants according to their flowering time. We found 134 out of the 145 identified loci for grain weight that were not associated with either plant phenology or plant height. The majority of loci uncovered here were novel, with favorable alleles widespread in Asian and African landraces providing opportunities for their incorporation into modern varieties through breeding. Using residual heterozygosity in lines from a nested association mapping population, we were able to rapidly develop near-isogenic lines for important target loci. One target locus on chromosome 6A contributed to higher grain weight, harvest index, thousand kernel weight, and grain number under drought and heat stress in field conditions consistent with allelic effects demonstrated in the genome-wide association study.

## Introduction

Bread wheat (*Triticum aestivum* L.) is one of the leading crops with an annual production of 730.9 million tons globally. However, the world's wheat consumption is expected to expand beyond production raising concerns about future food security (FAO, 2018; FAO, 2019). Wheat production is constrained by abiotic stresses such as drought and heat causing yield losses of up to 40% and 60% in the field, respectively (Zampieri et al., 2017). In many cropping regions these stresses occur simultaneously leading to almost total yield loss. For instance, wheat production in Mediterranean climate zones such as Australia, southern Europe and the northwest of the United States is largely based on dry land, characterized by drought in combination with high temperatures around anthesis and early grain filling (Izanloo et al., 2008; Schillinger et al., 2008; Gbegbelegbe et al., 2016; Toreti et al., 2019). At reproductive stages wheat yields are especially vulnerable with drought and heat stress reducing spikelet fertility, grain number, single grain weight, and grain filling duration (Prasad et al., 2011; Mahrookashani et al., 2017).

To reduce yield losses, the identification and incorporation of favorable alleles controlling grain yield and its components into cultivated varieties is crucial (Furbank and Tester, 2011). While bi-parental mapping populations include only a limited number of parental lines, genome-wide association studies (GWAS) are suitable for exploring larger and more diverse panels without the requirement to develop mapping populations (Zhu et al., 2008). To date, several quantitative trait loci (QTL) for yield and its components have been identified under drought, heat, and under combined drought and heat stress in field environments [reviewed in (Tricker et al., 2018)]. The identification of stress tolerance QTL in field conditions is, however, extremely difficult due to multigenic control, low heritability and large genotype × environment interactions, as well as the influence of several stresses at the same time (Fleury et al., 2010; Dolferus et al., 2011). In addition, most of the yield QTL found in these studies were associated with flowering time and plant height controlling genes, in particular photoperiod (*Ppd*), vernalization (*Vrn*), and reduced height (*Rht*) genes. The strong effect of flowering time and plant height on yield often masks the effects of other loci of smaller effects, limiting the detection of yield-regulating QTL. To minimize their cofounding effects, studies either account for flowering time and plant height by including them as covariates in statistical models or by calculating the residual effect of QTL unrelated to flowering time and plant height, but often find very few QTL (Lopes et al., 2015; Ogbonnaya et al., 2017; Mason et al., 2018).

The first genetic studies of wheat under a combination of drought and heat stress under controlled conditions were carried out by Aprile et al. (2013) in durum wheat and by Qaseem et al. (2018) in bread wheat. Experiments in pots under controlled conditions enable a more precise control of the environmental variables and the time of treatment. The disadvantage, however, is that results are not always reproducible in the field although they might be suitable for preliminary discovery and for avoiding high costs of field trials (Passioura, 2006; Izanloo et al., 2008). Qaseem et al. (2018) identified several QTL under well-watered and heat stressed conditions and one QTL under combined drought and heat stress for grain weight not linked to plant phenology.

In this study, we conducted a GWAS over 2 years using a diverse bread wheat collection consisting of 315 accessions. We measured yield components and traits previously hypothesized to be associated with combined drought and heat stress tolerance. Our aim was to identify novel QTL and alleles associated with combined drought and heat tolerance but independent from plant phenology. We used a semi-controlled pot system that allowed us to treat plants individually according to their flowering time. We developed near-isogenic lines (NILs) for one of the QTL and exposed these to combined drought and heat stress in field conditions to validate the effect of the locus.

## Materials and Methods

### Plant Material

For the GWAS, diversity panels composed of a total of 315 spring wheat accessions were evaluated in two independent experiments in 2016 and 2017. The two panels represented a reduced set of the panel described in Garcia et al. (2019) and differed in 110 accessions between both years due to identity issues, missing genotypic data, or late maturing types in 2016 (**Supplementary Table 1**). Accessions with uncertain identity were excluded from the analysis in 2016, resulting in a subset of 273 lines. Plants which flowered much later than the majority of the plants (i.e., seven and six plants in 2016 and 2017, respectively) were also excluded to avoid different treatment conditions due to the rising temperatures at the end of the experiments. Seeds for the 2016 panel were obtained from a pilot experiment in 2015 at Urrbrae (South Australia, Australia) grown in pots under well-watered conditions, whereas seeds for the 2017 panel were obtained from three different sources: a 2013 field trial at Urrbrae (South Australia, Australia; 293 accessions), a 2015 pilot experiment (16 accessions), and the Australian Grains Genebank (6 accessions).

Plant material for the validation of a target QTL identified during the GWAS in 2016, which was located on chromosome 6A, derived from an existing nested association mapping (NAM) population. Parents of the nested association mapping population formed part of the diversity panel and are listed in **Supplementary Table 1**. Twenty-eight BC1F4 families from the existing NAM population were available and used for screening for the target QTL. Four hundred and eighty recombinant inbred lines of the 20 families (BC1F4) were genotyped with the 90,000 single nucleotide polymorphism (SNP) marker “RAC875_s119505_143” (Wang et al., 2014), which was shown to have the strongest association within the QTL, to find lines that were heterozygous at this locus. Genotyping was performed using Kompetitive Allele Specific Polymerase Chain Reaction (KASP™) technology (LGC Limited, London, United Kingdom). KASP™ assays were designed in-house (**Supplementary Table 2**) and SNP and sequence information were obtained through the Diversity Among Wheat geNomes platform (Watson-Haigh et al., 2018). One hundred twenty-seven BC1F5 derived from single seed descendent of heterozygous recombinant inbred lines were genotyped using the selected marker to identify pairs of NILs carrying the allele from either the recurrent or diverse parent. Ten additional KASP SNP markers located on different chromosomes were used to validate the genetic background of the NILs and to select NIL pairs with similar phenology (**Supplementary Table 2**). In total, four NIL pairs (BC1F6) were identified. Three of the four NIL pairs derived from a cross between Gladius and a diverse donor (i.e., one from a cross with Taferstat, NIL pair 1, and two from a cross with Thori, NIL pairs 2 and 3), whereas one of the NIL pairs derived from a cross between Scout and Zilve (NIL pair 4).

### Plant Growth Conditions

The phenotyping for the GWAS was carried out in pots under semi-controlled conditions in a polytunnel facility at the University of Adelaide (Urrbrae, South Australia, Australia, 35° S 139° E) from May to November in 2016 and 2017. A split-plot design with three biological replications per treatment surrounded by a line of border pots was adopted in both years (**Supplementary Figure 1**). Plants were randomized over three blocks (i.e., one replicate per block) and randomized differently in each year to avoid that genotypes were located at the same spot as the year before. The polytunnel facility consisted of a main area with tables at the back to dry the pots down for the drought treatment and an adjacent heat chamber for the heat treatment. Single plants were grown in pots filled with 0.5 kg of a substrate mix of clay-loam, sand, and coco peat in a 1:1:1 ratio and supplemented with a basal, slow-release fertilizer. Plants were additionally fertilized at tillering (All-Purpose Soluble Fertilizer, Hortico, Australia) and heading (Trace Element Soluble Powder, Manutec, Australia) in 2016 and at early booting in 2017 (All-Purpose Soluble Fertilizer, Hortico, Australia). Pesticides were used for an adequate pest and disease control. Temperature and relative humidity were recorded throughout both experiments in the main and in the heat area. Temperature was monitored at 10 minutes intervals with the Hobo Monitoring Station Data Logger RX3000 (Onset Computer Corporation, United States). Sensors were installed at 10 cm above pot level at the beginning of each experiment and adjusted fortnightly to plant canopy height. Relative humidity was recorded every 10 minutes in the heat chamber with a hobo sensor and in the main area with four dataloggers (model KG100, Kongin, China), placed at each of the corners of main area at pot level. Soil moisture was monitored on the last day of treatment. Plants were supplied with sufficient water from sowing to anthesis. The primary tiller of each plant was tagged at anthesis. At 3 days after anthesis, plants were subjected to either drought treatment (D): irrigation withheld for 6 days; or combined drought and heat (DH) treatment: irrigation withheld for 6 days and 35/25°C day/night from the fourth day of D treatment on. After 6 days of treatment, plants were re-irrigated and kept under well-watered conditions until the end of the experiment.

NILs were grown in micro-plots under semi-controlled conditions in a polytunnel facility at the University of Adelaide (Urrbrae, South Australia, Australia) in 2018. A randomized block design with three biological replications was implemented. NILs of the same pair were kept next to each other to minimize spatial heterogeneity. A border around each plot was planted to reduce interplot competition (Rebetzke et al., 2014). For each plot, two rows of eight seeds were sown with a plant density of 190 plants m-2 and a plot size of 20 x 42 cm. Sowing was later (20th of June) than the normal commercial sowing time in South Australia (April/May) to assure temperatures above 35°C during anthesis and grain filling. Plants which did not germinate by the 11th July 2018 were replaced by 6-days old seedlings grown in petri dishes. Two soil probes (Measurement Engineering Australia, Australia), one at 10 and one at 40 cm soil depth, were installed in each block to measure the soil water potential every 10 minutes during the experiment. Soil probes were placed between the same NIL pairs in each block to prevent differences in soil water potential caused by different genotypes. Temperature and relative humidity were recorded at 10 minutes intervals by installing one datalogger in the middle of each block. Plants were fertilized at 5-leaf stage with 50 kg/ha nitrogen (Urea, Richgro, Australia) and 10 kg/ha phosphate (Superphosphate, Richgro, Australia). A second nitrogen (30 kg/ha, Urea, Richgro, Australia) application was performed at the end of stem elongation. Pesticides were applied according to usual field practices. Plants were regularly irrigated using a drip-irrigation system maintaining the soil water potential below −100 kPA. The Zadoks' stage of each plot was recorded three times a week. At Zadoks' stage 39 (i.e., the flag leaf collar was visible in more than 50% of the plots) irrigation was stopped to impose severe drought stress during early grain filling. Plots were lightly re-irrigated three times during the course of the experiment (i.e., drip irrigation for 11 minutes, corresponding to 17 mm of rain fall) the day after all six soil sensors marked −633 kPa to mimic cyclic drought events. To subject plants to a combination of drought and heat stress during early grain filling, the polytunnel was partly closed at Zadoks' stage 65 (i.e., anthesis half complete in more than 50% of the plots) for three weeks.

### Phenotypic Data

Morphological, physiological, and grain traits were measured in the pot experiment for all three replicates under both treatments. Days to anthesis was defined as the time from sowing until the first visible anther of the primary tiller. The leaf water potential of the second leaf of the primary tiller was measured on the fifth day of treatment. Leaf samples were collected daily between 8:30 and 11:00 am and placed into a plastic cup, sealed with parafilm, and kept in a moist bag until they were measured with a water potential meter (WP4C, Meter Group, United States) in precise mode for 5 minutes. A self-calibrating chlorophyll meter (SPAD 502 Plus, Spectrum Technologies, United States) was used to measure the chlorophyll content in the center of the flag leaf at 9 days after anthesis as an average of three measurements. At physiological maturity, plant height of the primary tiller was measured from the base of the plant to the tip of the spike excluding awns. Spike length of the primary tiller was determined by measuring the distance between the base of first rachis to the tip of the last spikelet without awns. Number of spikes per plant and total above-ground biomass, including leaves, stem, and spikes of all tillers, were recorded. Spikes of the primary tiller and other tillers were kept separate and threshed by hand. Grain screenings were obtained for the primary tiller and the whole plant with a wheat grain sieve (2.0 mm, Graintec, Australia) and determined as the percentage of the ratio between small grain weight (i.e., non-filled grains) and total grain weight. Number of grains of > 2.0 mm of size (i.e., filled grains) were counted for primary tiller and plant. Grain weight was determined as the weight of grains > 2.0 mm in primary tiller and plant. Single grain weight was calculated for both primary tiller and whole plant as the ratio between grain weight and the number of grains. Harvest index was estimated by dividing grain weight of the whole plant by the above-ground biomass.

In 2018, days to anthesis was defined as the time from sowing until more than half of the plants in a plot reached Zadoks' stage 65 (Zadoks et al., 1974). Plant height and spike length of the primary tiller (i.e., the tallest tiller of each plant) of five randomly chosen plants of each plot were measured at physiological maturity as described above. Spikes of the primary tiller of the five selected plants were harvested separately from the rest of the plants of each plot to potentially increase the statistical power due to an increased sample size. Single spikes and whole plants per plot were oven-dried in a paper bag at 37°C for 10 days. Subsequently, number of spikes per plot and total above-ground biomass per plot including all spikes were measured. Single spikes were threshed by hand, while the rest of the spikes were threshed with a conventional threshing machine. Both parts were sieved separately by hand (wheat grain sieve 2.0 mm, Graintec, Australia). For the single spikes, grain weight, grain number, and single grain weight of grains > 2.0 mm and screenings were determined as described before. Traits per plot included grain weight, grain number, screenings, and thousand kernel weight. Harvest index was calculated as the ratio between grain weight per plot and above-ground biomass.

### Genotyping and Population Structure of Diversity Panels

Genotyping and the population structure analysis of the original diversity panel are described in Garcia et al. (2019). A total of 563 accessions were genotyped using the wheat iSelect 90K SNP genotyping array (Wang et al., 2014). After filtering for SNPs with minor allele frequency of < 5% and missing values > 5%, 30,533 unique, high-quality SNPs remained and were used for association analyses. Additionally, the genotypic data of ten markers associated with genes known to affect plant phenology (*Ppd-A1*, *Ppd-B1*, *Ppd-D1*; *Vrn-A1*, *Vrn-D1*), plant height (*Rht-B1*, *Rht-D1*, *Rht24*), and grain weight (*TaGW2-6A*, *TaGW2-6B*) were included.

### Statistical Analysis of Phenotypic Data

Adjusted means (BLUEs) were calculated for each trait under D and DH treatment in both GWAS using the R package ASReml (Butler et al., 2009), fitting accessions and treatments as fixed effects and factors relating to the experimental design as random effects. Days to anthesis was significantly associated with all traits. Predicted means were therefore calculated twice as previously done in durum wheat by Sukumaran et al. (2018): i) without including days to anthesis as a covariate (i.e., not adjusted) and ii) including days to anthesis as a covariate (i.e., adjusted). To assess the heat response under drought of each genotype, a ratio of the predicted, non-adjusted means under DH divided by the predicted, non-adjusted means under D was calculated for all traits, except for days to anthesis. The outputs for D (adjusted and non-adjusted means), DH (adjusted and non-adjusted means), and the ratio were used for genome-wide association analysis. The heritability of each trait under D and DH was calculated according to Cullis et al. (2006) using a secondary model with accessions as random effects. Two-way analysis of variance and Tukey's HSD test were carried out to test for significant differences between non-adjusted means. Pearson correlation coefficients were estimated to investigate the relationship among traits and represented in a principal component analysis biplot.

Means for traits per spike and per plot in 2018 were predicted for each NIL pair separately using ASReml. The two NILs of each pair were implemented as fixed effects and factors relating to the experimental design as random effects. Days to anthesis, defined as the days from sowing until Zadoks' stage 65, was included as fixed effect if significantly associated with the trait, which was the case for NIL pair 1 for biomass, grain weight and grain number per plot, NIL pair 4 for grain number, single grain weight and screenings per spike, and NIL pair 2 and 3 for plant height. Significant differences among NIL pairs were estimated conducting Tukey's HSD test. Correlations between traits were calculated using Pearson coefficients.

### Genome Wide Association Analysis

Genome-wide association analysis was performed with the adjusted means for each of the two treatments and the ratio in both years. We used the compressed mixed linear model of Zhang et al. (2010) implemented in the R package “Genomic Association and Prediction Integrated Tool” (GAPIT) (Lipka et al., 2012) and accounted for population structure and genetic relatedness. A model selection procedure was run to determine the optimal number of principal components per trait to be included in the association analysis, with a maximum of four principal components. A two-level false discovery rates (FDR) (Benjamini and Hochberg, 1995) of 0.05 and 0.20 was used as threshold for declaring significant MTA. FDR adjusted p-values were obtained from the GAPIT output files. The difference between the variation explained by the MTA with and without the strongest associated SNP was used to estimate the allelic effect of each MTA (Sun et al., 2010). The order of significant and indicative MTA was determined based on the wheat consensus map of Wang et al. (2014). The position on the physical map was determined by aligning the sequences of the markers to the RefSeq v1.0 (IWGSC, 2018), using BLASTN with an e-value cut-off of 10^−5^. MTA which could not be assigned to a chromosome were not considered. The intervals for the QTL were defined by comparing the position of the significant markers on the consensus and physical maps. Map graphics were drawn using the R package ggplot 2 (Wickham, 2016).

## Results

### Effects of Drought or Combined Drought and Heat Stress on Phenotypic Traits

Two treatments, drought (D), and combined drought and heat stress (DH) were imposed 3 days after anthesis of each individual plant. Plants were subjected to D by withholding water for 6 days while plants in the DH treatment were subjected to the same treatment for 3 days and then moved to a heat chamber for another 3 days without watering. This resulted in a severe post-anthesis drought stress of 3.1% average soil water content, coupled, in plants under DH treatment, with high temperature stress of 31.0/23.4°C day/night in 2016 and 32.2/24.4°C day/night in 2017 (**Supplementary Figure 2**). Weather conditions for both years were similar with average temperatures of 17.3/11.6°C day/night in 2016 and 16.8/13.2°C day/night in 2017 in the main area outside the heat chamber. Maximum temperatures were slightly higher in the main area in 2016 with 25 days above 30°C in comparison to 8 days in 2017. On average, relative humidity reached 69.0% in 2016 and 68.9% in 2017 in the main area and 50.7% in 2016 and 44.1% in 2017 in the heat chamber.

Heritability estimates (H^2^) were similar in both years ranging from 40.9% for grain weight of primary tiller to 99.6% for the number of days to anthesis (**Table 1**). Moderate H^2^ were found for grain traits and harvest index under DH (40.9–66.3%) while under D, H^2^ were high (73.7–91.3%). The lower H^2^ under DH is probably due to an increased number of plants with zero grain weight caused by severe stress.

**Table 1:** Predicted means, minimum, and maximum values as well as heritability (H^2^) under drought and combined drought and heat stress in 2016-2017.

Trait	Treatment	2016				2017			
		Mean	Min	Max	H^2^ (%)	Mean	Min	Max	H^2^ (%)
Days to anthesis	Pre-treatment	121.8	91.6	186.2	99.6	119.2	95.7	162.8	98.7
Leaf water potential (MPa)	Drought	-6.5	-65.2	-2.0	85.7	-13.2	-77.3	-1.5	78.4
	Drought & Heat	-21.3	-171.4	-3.4	84.3	-45.6	-132.8	-3.5	84.3
Chlorophyll content	Drought	26.4	4.2	55.8	76.7	39.5	9.8	63.1	75.9
	Drought & Heat	24.3	1.2	60.7	79.8	43.5	12.3	73.6	75.9
Number of spikes	Drought	2.7	1.2	5.1	57.0	3.3	1.0	6.0	68.3
	Drought & Heat	2.8	1.5	5.4	56.7	3.3	1.4	6.5	70.4
Spike length (cm)	Drought	10.4	5.2	13.9	73.8	11.5	4.5	16.7	91.2
	Drought & Heat	10.6	4.7	14.3	75.5	11.6	4.9	18.7	90.7
Plant height (cm)	Drought	104.2	50.9	149.5	92.6	114.1	58.8	173.0	94.8
	Drought & Heat	104.2	51.5	155.1	92.9	114.5	56.3	164.5	95.7
Biomass (g)	Drought	12.7	4.4	31.0	77.5	17.6	4.5	42.5	79.0
	Drought & Heat	10.9	3.3	24.7	87.3	14.0	3.1	40.4	93.3
Screening per primary tiller (% small grain weight)	Drought	10.1	0.0	100.0	78.7	15.1	0.0	100.0	74.0
	Drought & Heat	83.3	7.63	100.00	42.4	90.6	0.0	100.0	63.2
Screening per plant (% small grain weight)	Drought	9.1	0.0	100.0	82.6	14.3	0.0	100.0	74.3
	Drought & Heat	80.3	6.2	100.0	50.1	89.8	0.0	100.0	63.7
Number of grains per primary tiller	Drought	41.8	0.0	72.4	84.4	39.9	0.0	80.2	76.9
	Drought & Heat	6.7	0.0	47.3	43.2	3.4	0.0	33.0	57.7
Number of grains per plant	Drought	88.0	0.0	155.3	77.6	104.6	3.3	176.1	73.7
	Drought & Heat	13.4	0.0	100.6	48.6	5.8	0.0	67.0	57.0
Single grain weight per primary tiller (mg)	Drought	42.3	0.0	64.7	83.1	40.5	0.0	67.7	84.2
	Drought & Heat	7.2	0.0	42.8	41.9	4.1	0.0	51.3	66.3
Single grain weight per plant (mg)	Drought	41.7	0.0	70.0	81.3	39.7	0.0	67.1	82.5
	Drought & Heat	9.4	0.0	38.1	50.2	4.6	0.0	47.2	66.2
Grain weight per primary tiller (g)	Drought	1.90	0.00	3.81	84.0	1.80	0.00	3.86	81.3
	Drought & Heat	0.20	0.00	1.34	40.9	0.10	0.00	1.68	63.8
Grain weight per plant (g)	Drought	3.79	0.00	6.55	82.4	4.50	0.11	9.00	78.3
	Drought & Heat	0.38	0.00	2.38	44.6	0.17	0.00	3.28	63.1
Harvest Index	Drought	0.32	0.00	0.51	91.3	0.26	0.00	0.47	84.7
	Drought & Heat	0.04	0.00	0.24	45.1	0.01	0.00	0.23	64.6

Under DH, grain weight, single grain weight, and the number of grains > 2.0 mm were significantly lower (p ≤ 0.001) compared to D with similar results in primary tillers and whole plants (**Table 1**, **Supplementary Figure 3**). Screenings significantly increased (p ≤ 0.001) under DH compared to D. Grain weight was the trait most severely affected by DH with an average reduction of 92.1% in primary tillers and 93.1% in whole plants across years, followed by grain number and single grain weight with average reductions of 87.8–89.6% and 82.9–86.5%, respectively. Screenings was the least affected grain trait, increasing on average by 71.2–75.6%. Similar to grain weight, leaf water potential, biomass, and harvest index were significantly reduced by DH compared to D in both years (p ≤ 0.001), whereas no significant effect was observed for plant height and spike length in 2016 and 2017 and for spike number and chlorophyll content in 2017.

Grain weight of the primary tiller and whole plant did not differ under DH between the years, while grain components (i.e., grain number, single grain weight, and screenings) were significantly more affected by DH in 2017 compared to 2016 (p ≤ 0.001) in both primary tillers and whole plants. D had a similar effect in both years on grain weight, grain number, and single grain weight per primary tiller but had a significantly higher impact on grain weight and grain number per plant in 2016 compared to 2017 (p ≤ 0.001). In contrast, screenings per primary tiller and plant were more affected in 2017 than in 2016 (p ≤ 0.05 and p ≤ 0.001, respectively). Number of days to anthesis was reduced by 23 days in 2017 compared to 2016 due to the replacement of late maturing types. A narrowed flowering time window would suggest a decreased exposure to higher temperatures, as they often occur toward the end of the season, and might therefore explain the higher number of grains and grain weight per plant under D in 2017. Differences in grain components between years under DH were, in contrast, most likely caused by the overall 1°C increase in temperature in the heat chamber in 2017.

**Figure 1:**
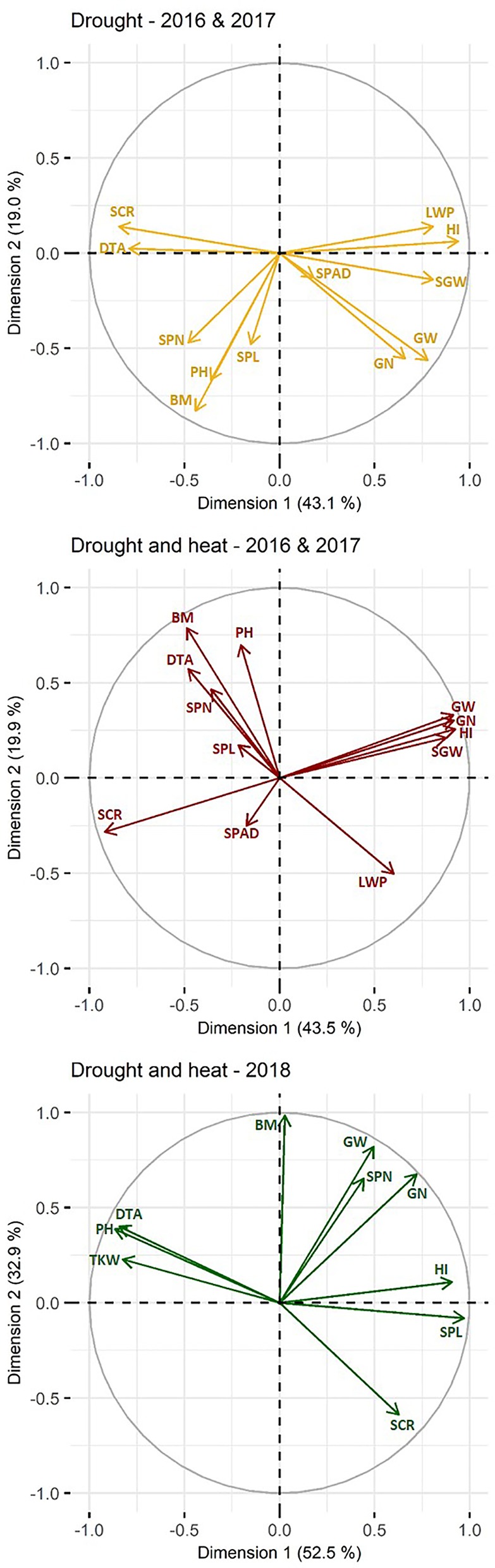
Principal component analysis biplot of correlations among traits. Traits studied in 2016 and 2017 under drought are marked in yellow, under combined drought and heat in red and traits studied in 2018 under combined drought and heat are marked in green. Positively correlated traits are grouped together, whereas negatively correlated traits are positioned on opposite quadrants. The distance between traits and the plot origin indicates the quality of representation of the trait within the principle component analysis (i.e., the further away, the better represented). For simplicity, grain traits measured in 2016 and 2017 are only given for whole plant and grain traits measured in 2018 are given per plot. DTA, days to anthesis; HI, harvest index; LWP, leaf water potential; SPAD, chlorophyll content; SPN, number of spikes; SPL, spike length; PH, plant height; BM, biomass; SCR, screenings; GN, grain number; SGW, single grain weight; SW, grain weight; single grain weight per spike; TKW, thousand kernel weight.

Phenotypic correlations (R^2^) between traits under D and DH treatment are presented in **Supplementary Table 3** and **Figure 1**. Under D and DH, 43.1–43.5% and 19.0–19.9% of the variation is explained by the first and second dimension, respectively, explaining thus more than (62.1–63.4%) half of the variation (**Figure 1**). All traits, except chlorophyll content and spike length were well represented by the principal component analysis. Adjusted means of grain weight, screenings, grain number, and single grain weight of the primary tiller were highly correlated with those of whole plants under D and DH with R^2^ between 0.75 and 0.95 (p ≤ 0.001). Under D and DH, grain weight had a significant (p ≤ 0.001) and positive correlation with leaf water potential, grain number, single grain weight, and harvest index. In contrast, days to anthesis, spike number, and screenings were negatively associated with grain weight in both treatments.

### Genome-Wide Association Studies

**Figure 2:**
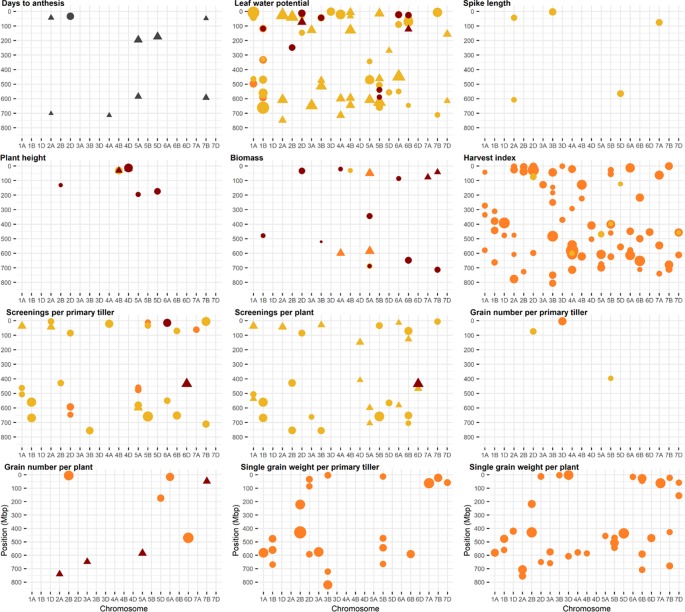
Physical position of marker trait associations (MTA). A false discovery rate of 0.20 was set as threshold. Physical positions are based on IWGSC RefSeq v1.0. Traits in yellow represent MTA identified under drought, red represents MTA identified under combined drought and heat, and orange represents MTA identified for the ratio (i.e., heat response under drought). Markers associated with days to anthesis are in grey. MTA identified in 2016 are represented as a circle and MTA identified in 2017 as triangle. The size of the circle and triangle corresponds to the allelic effect of each MTA.

Identified markers and their corresponding QTL are shown in **Figure 2**, except for QTL for grain weight which are summarized in **Table 2**. Details of QTL including number of associated markers, position on genetic and physical map, and allelic effect can be found in **Supplementary Table 4**. Examples of Manhattan plots and Q-Q plots are given in **Supplementary Figure 4**. A total of 256 and an additional 216 QTL were identified using a FDR of 0.05 and 0.20, respectively, representing an average of 5 QTL per trait, treatment, and year with an average QTL interval of 1.2 Mbp. QTL were found on all chromosomes with most QTL located on chromosomes 3B, 5A, 5B, and 6B. Of the 472 QTL, 133 QTL were associated with D, 53 with DH, and 276 were found for the heat response under drought. Three hundred twenty-seven QTL co-located with QTL across more than one treatment, of which 81 QTL were pleiotropic for D, DH, and the heat response under drought. No QTL for leaf chlorophyll content were found.

**Table 2:** QTL controlling grain weight.

Chr	QTL	Trait	Treatment	Year	Position (cM)	Position (bp)	Allelic effect (%)	Traits with same QTL location (Treatment)
**QTL for combined drought and heat**								
3A	*QGWt.adh-3A QGWp.adh-3A*	GWt GWp	DH	2017	347.9-349.3	647,474,241-647,508,573	3.9-4.0	LWP (D), GNp (DH), HI (DH)
3B	*QGWp.adh-3B.1*	GWp	DH	2017	56.4	14,985,191-14,985,392	3.7	HI (DH)
3B	*QGWp.adh-3B.2*	GWp	DH	2017	119.8	26,650,089-29,356,945	3.8	SCRp (D), GWt (Ratio), HI (DH)
5B	*QGWp.adh-5B*	GWp	DH	2017	401.5-403.3	622,066,480-623,585,489	3.5	LWP (D), GWt (Ratio), HI (Ratio)
6B	*QGWp.adh-6B*	GWp	DH	2017	na	71,040,399-71,495,726	3.3	LWP (D), SCRt (D), SCRp (D)
								
**QTL for drought**								
2D	*QGWt.adr-2D*	GWt	D	2017	109.7-114.4	73,570,876-78,765,908	4.1-4.7	GNt (D), HI (D)
2D	*QGWp.adr-2D*	GWp	D	2016	133.2	146,305,492-146,305,593	7.9	LWP (D)
								
**QTL for the heat response**								
1A	*QGWt.ara-1A.6*	GWt	Ratio	2016, 2017	431.5-435.2	579,299,114-581,438,572	5.0-8.7	SGWt (Ratio), SGWp (Ratio), HI (Ratio)
3D	*QGWt.ara-3D.1*	GWt	Ratio	2016, 2017	na	1,698,974-4,394,598	5.2-9.0	LWP (D), GNt (Ratio), SGWp (Ratio), HI (Ratio)
6B	*QGWt.ara-6B.7 QGWp.ara-6B.3*	GWt GWp	Ratio	2016, 2017	375.2-388.2	705,384,526-712,346,484	4.8-5.8	SCRp (D), SGWp (Ratio), HI (Ratio)
6D	*QGWt.ara-6D.2 QGWp.ara-6D*	GWt GWp	Ratio	2016, 2017	330.3	461,924,775-471,922,386	5.0-14.0	SCRp (D), GNp (Ratio), SGWp (Ratio), HI (D)
7A	*QGWt.ara-7A.1*	GWt	Ratio	2016, 2017	262	62,528,244-63,443,715	11.8-14.7	SCRt (Ratio), SGWt (Ratio), SGWp (Ratio), HI (D, Ratio)
7B	*QGWt.ara-7B.1 QGWp.ara-7B*	GWt GWp	Ratio	2016, 2017	61.4-87.3	1,258,258-6,393,796	4.6-10.8	LWP (D), SCRt (D), SCRp (D), HI (Ratio)
								
**Stable QTL under drought and heat stress**								
3B	*QGWt.ara-3B.3*	GWt	Ratio	2017	na	44,283,482-44,283,582	9.2	LWP (D, DH), SPN (Ratio), HI (Ratio)
4A	*QGWt.ara-4A.1 QGWp.ara-4A*	GWt GWp	Ratio	2017	na	21,063,714-21,635,963	4.4-9.1	LWP (D), BM (DH), SCRt (D), HI (Ratio)
5B	*QGWt.ara-5B.6*	GWt	Ratio	2017	242.8-247.3	539,296,240-559,072,690	3.6-3.7	LWP (D, DH), SGWt (Ratio), SGWp (Ratio)
6A	*QGWt.ara-6A.1 QGWp.ara-6A*	GWt GWp	Ratio	2016, 2017	77.7-80.1	12,837,679-16,232,972	4.8-11.8	SCRt (DH), SCRp (D), GNp (Ratio), SGWp (Ratio), HI (Ratio)
6A	*QGWt.ara-6A.3*	GWt	Ratio	2017	178.6	85,756,394-99,014,241	3.6	LWP (D), BM (DH)
6B	*QGWt.ara-6B.6*	GWt	Ratio	2017	259.8	646,565,102-652,374,782	12.4-15.4	LWP (D), BM (DH), SCRt (D), SCRp (D), HI (Ratio)
7B	*QGWt.ara-7B.6*	GWt	Ratio	2017	463.6	701,871,740-712,736,264	5.6-5.8	LWP (D), BM (D, DH), SCRt (D), HI (Ratio)

Position in base pairs corresponds to RefSeq v1.0. (IWGSC, 2018). Positions in centimorgan are according to the consensus map from Wang et al. (2014). bp, base pairs; BM, biomass; Chr, chromosome; cM, centimorgan; D, drought; DH, combined drought and heat; HI, harvest index; LWP, leaf water potential; SCRt, screenings per primary tiller; SCRp, screenings per plant; GNt, number of grains per primary tiller; GNp, grain number per plant; SPN, number of spikes; SGWt, single grain weight per primary tiller; SGWp, single grain weight per plant; GWt, grain weight per primary tiller; GWp, grain weight per plant.

#### QTL for Flowering Time and Plant Height

The strongest locus for days to anthesis was the known photoperiod sensitive locus *Ppd-D1* (*QDTA.aco-2D*) on chromosome 2D explaining 3.5–6.3% of the phenotypic variation. *Ppd-D1* was also associated with biomass under DH in 2016 and co-located with two QTL for single grain weight per primary tiller for the heat response under drought (*QSGWt.ara-2D*) and leaf water potential under D (*QLWP.adr-2D.2*). Further QTL for days to anthesis were found on chromosomes 2A, 4A, 5A, 5D, and 7B of which seven co-located with QTL for grain weight. QTL on chromosome 5A (*QDTA.aco-5A.1*) and 5D (*QDTA.aco-5D*) co-located also with QTL for plant height (*QPH.adh-5A*, *QPH.adh-5D*). The major loci associated with plant height were *Rht-B1* (*QPH.adr-4B*, *QPH.adh-4B*) and *Rht-D1* (*QPH.adr-4D*, *QPH.adh-4D*) on chromosome 4B and 4D, respectively. Both QTL appeared under D and DH and in both years. Another QTL for plant height (*QPH.adh-2B*) was identified under DH in 2016 located on chromosome 2B. None of the five QTL for plant height was associated with grain weight components.

#### QTL for Combined Drought and Heat

QTL under DH explained, on average, 4.4% of the phenotypic variation with QTL for plant height having the largest allelic effect (8.7%), followed by QTL for screenings per primary tiller and plant (6.2–7.6%) and for leaf water potential (6.4%). QTL for grain weight and grain number explained 3.3–5.2% and 3.9–4.8% of the phenotypic variation, whereas QTL for biomass accounted for the smallest phenotypic variation (2.4%). The maximum allelic effect of QTL for harvest index under DH was 4.9%.

Six QTL for grain weight per primary tiller and per plant independent from flowering time were identified under DH on chromosome 3A, 3B, 5B, and 7B using a FDR of 0.20 (**Table 2**). The strongest QTL was detected on the long arm of chromosome 3A. QTL for grain weight for the heat response co-located with two of the QTL for grain weight under DH on chromosome 3B (*QGWp.adh-3B.2*) and 5B (*QGWp.adh-5B*). QTL for harvest index, leaf water potential, screenings, and grain number co-located with seven, five, three, and two of the eight QTL for grain weight, respectively. The positive allele of the QTL for grain weight on 3B (*QGWp.adh-3B.2*) was mostly found in Asian landraces. Breeding lines from the International Maize and Wheat Improvement Center (CIMMYT) in Mexico and Australia carried mostly the positive allele for QTL located on chromosome 3A (*QGWp.adh-3A*), whereas the positive allele of the second 3B QTL (*QGWp.adh-3B.1*) was predominantly found in the North American germplasm. The positive alleles of *QGWp.adh-5B* and *QGWp.adh-6B* were common among all accessions and wheat types, but less common in Asian accessions and landraces.

#### QTL Under Drought

Under D, the identified QTL explained on average 5.9% of the phenotypic variation with the strongest QTL associated with leaf water potential accounting for 16.9% of the variation. Allelic effects at QTL for biomass (3.4–4.6%) explained the least phenotypic variation. The allelic effects of QTL for the yield component traits grain weight, grain number, and screenings ranged from 3.5 to 12.0% with the highest percentage of phenotypic variation explained for screenings per primary tiller.

Two QTL for grain weight per primary tiller and plant under D were identified with a FDR of 0.20 on chromosome 2D (*QGWt.adr-2D*, *QGWp.adr-2D*) accounting for a maximum of 4.7 and 7.9% of the phenotypic variation. Both QTL clustered together with QTL for leaf water potential. *QGWt.adr-2D* also coincided with QTL for grain number and harvest index. The positive alleles for *QGWt.adr-2D* and *QGWp.adr-2D* were common in breeding lines from CIMMYT and Australia.

#### QTL for the Heat Response Under Drought

QTL for the heat response under drought elucidated an average of 7.3% of the phenotypic variation. QTL for biomass, spike number, and screenings accounted for the least phenotypic variation (4.7–5.9, 5.4–6.3, and 4.1–6.3%, respectively), while QTL for grain weight explained most of the phenotypic variation (3.6–21.2%). The allelic effects of QTL for single grain weight, grain number, and harvest index ranged from 3.5 to 19.0%. Allelic effects were potentially inflated by the calculation of the ratio. However, the use of a ratio also increased the statistical power allowing us to detect a larger number of QTL and a strong target QTL on chromosome 6A.

Using the ratio between environments, a total of 88 genomic regions were associated with grain weight per primary tiller and per plant with a FDR of 0.05. The most important pleiotropic regions were located on chromosome 1A (*QGWt.ara-1A.6*), 3D (*QGWt.ara-3D.1*), 6B (*QGWt.ara-6B.7*, *QGWp.ara-6B.3*), 6D (*QGWt.ara-6D.2*, *QGWp.ara-6D*), 7A (*QGWt.ara-7A.1*), and 7B (*QGWt.ara-7B.1*, *QGWp.ara-7B*) with chromosome 7A having the strongest allelic effect on grain weight (11.8–14.7%). All six regions were associated with harvest index and five of the six regions included QTL for single grain weight (i.e., all except the one on chromosome 7B). QTL for screenings were located within four (on chromosome 6B, 6D, 7A, and 7B) genomic regions. Grain number and leaf water potential were associated with two of the six genomic regions on chromosomes 3D and 6D and on chromosomes 3D and 7B, respectively. QTL for grain weight appeared in both years in all genomic regions and positive alleles were predominantly found in Asian and African landraces.

#### Stable QTL Under Drought and Heat Stress

Fourteen genomic regions independent from plant phenology were significantly associated with the two treatments and the heat response under drought of which half were also stable across years. The seven genomic regions were located on chromosomes 3B, 4A, 5B, 6A, 6B, and 7B and were associated with grain weight per primary tiller and plant for the heat response under drought. Six of the nine QTL for grain weight (*QGWt.ara-3B.3*, *QGWt.ara-4A.1*, *QGWp.ara-4A*, *QGWp.ara-6A*, *QGWt.ara-6B.6*, *QGWt.ara-7B.6*) were detected with an FDR of 0.05 and allelic effects ranged from 3.6 to 15.4% with the strongest QTL located on chromosome 6B. The positive alleles of *QGWt.ara-3B.3*, *QGWt.ara-4A.1*, *QGWp.ara-4A*, *QGWt.ara-6A.1*, *QGWp.ara-6A*, *QGWt.ara-6A.3*, *QGWt.ara-6B.6*, and *QGWt.ara-7B.6* were common in Asian and African landraces, whereas the positive allele of *QGWt.ara-5B.6* was mostly present in North American breeding lines. Six of the seven genomic regions were also associated with leaf water potential, four with biomass, harvest index, and screenings, two with single grain weight and one with grain number and spike number per plant.

### Validation of Candidate QTL in Near Isogenic Lines

NILs were developed for the validation of a target QTL in semi-controlled field plots. The selected QTL on chromosome 6A (*QGWt.ara-6A.1*, *QGWp.ara-6A*) (**Table 2**) belonged to one of the seven genomic regions which were stable across years, traits, and treatments. It was also the only genomic region which was associated with a grain weight component under DH in 2016 (*QSCRt.adh-6A*) and co-located with QTL for grain number per plant, single grain weight per plant, and plant and harvest index (*QGNp.ara-6A*, *QSGWp.ara-6A*, *QHI.ara-6A.1*). In addition, the positive alleles of *QGWt.ara-6A.1*, *QGWp.ara-6A*, *QGNp.ara-6A*, *QSGWp.ara-6A*, and *QHI.ara-6A.1* were predominantly in African and Asian landraces and not present in most breeding lines, representing a potential candidate for the integration of novel alleles in current breeding programs. Plants of the four NIL pairs at the target region on chromosome 6A were exposed to high temperature stress and cyclic drought (**Supplementary Figure 2**) with relative humidity reaching 56.8%.

**Figure 3:**
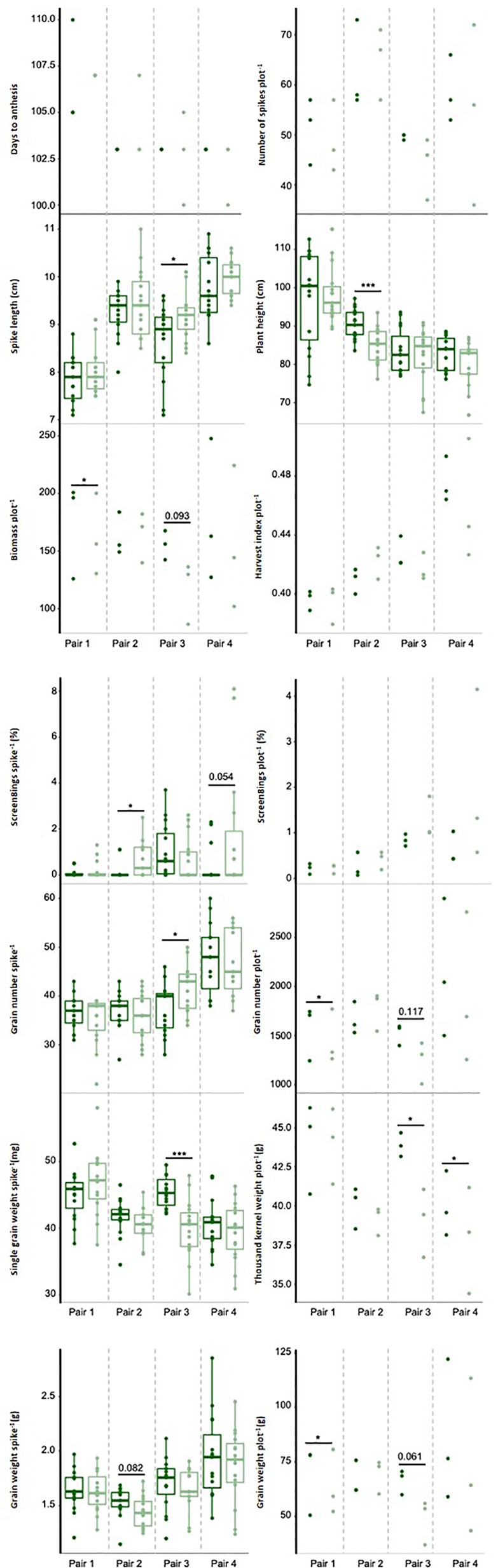
Phenotypic traits measured in 2018 under combined drought and heat stress. Near isogenic lines (NILs) of the same pair are next to each other carrying either the exotic (dark green) or non-exotic (light green) allele at the target region. Dots represent raw values of NILs. * and *** indicate statistical significance at p ≤ 0.05, p ≤ 0.01, and p ≤ 0.001, respectively, based on Tukey's HSD test. Numbers above black line represent p-values which are marginally significant.

Descriptive statistics of phenotypic data are given in **Supplementary Table 5** and represented in **Figure 3**. Correlations between traits are shown in **Supplementary Table 3** and **Figure 1**. Both dimensions of the principal component analysis explained 85.0% of the variation and all traits were well presented. A strong correlation between spike- (i.e., average of five spikes) and plot-based measurements was found for screenings (0.73, p ≤ 0.05) as well as single grain weight and thousand kernel weight (0.98, p ≤ 0.001). Correlations between spikes and plots for grain number and grain weight were only moderate (0.50–0.56) and insignificant. Among the plot-based measurements, grain weight showed the highest positive correlation with grain number (0.95, p ≤ 0.001), followed by biomass with a correlation of 0.85 (p ≤ 0.01). Flowering time and plant height were similar across all NILs with no differences within NIL pairs, except for NIL pair 2 with an average difference of 6 cm in plant height. Nevertheless, the increase in plant height was not significantly associated with the increase in grain weight in NIL pair 2.

Consistent with the findings from the GWAS in 2016, grain weight per spike and per plot, grain number per plot, and harvest index were increased under DH by the allele from the exotic parents (i.e., Taferstat, Thori, or Zilve) in at least three of the four NIL pairs, whereas screenings per spike and per plot were increased by the non-exotic allele in three NIL pairs. Increases in grain weight per plot ranged between 9.0% (NIL pair 1, p = 0.038) to 26.4% in NIL pair 3 (p = 0.061), followed by grain number per plot with an increase of 8.7 to 18.2% (NIL pair 1 p = 0.012 and NIL pair 3 p = 0.117, respectively). Screenings per spike showed the smallest impact of the QTL with 0.1 to 0.6% (NIL pair 4 p = 0.054 and NIL pair 2 p = 0.012, respectively). Screenings in both GWAS and QTL validation were not normally distributed. Nevertheless, findings from the QTL validation coincided with results from the GWAS in 2016, indicating that the results were sufficiently explained by a linear model. The exotic allele also increased single grain weight, thousand kernel weight, spike number, and biomass with a significant increase in single grain weight in NIL pair 3 (p ≤ 0.001, 12.2%), thousand kernel weight in NIL pair 3 and 4 (p = 0.023, 2.9–11.0%), and biomass in NIL pair 1 (p = 0.013) and marginally significant differences in NIL pair 3 (p = 0.093). In contrast, the non-exotic allele increased spike length with significant differences found for NIL pair 3 (p = 0.041). The only inconsistency with the previous results in pot-based GWAS experiments was the significant, positive effect of the non-exotic allele on grain number per spike in one of the four NIL pairs. The QTL interval contains 68 high-confidence genes in the Chinese Spring reference genome (**Supplementary Table 6**) but the gene content might differ in the parents of the NILs.

## Discussion

Drought and heat constrain wheat yields in many wheat growing regions of the world and their combined effect can cause severe yield losses (Toreti et al., 2019). A comprehensive understanding of the traits and loci conferring drought and heat tolerance will be therefore critical for future crop production in terms of climate change and climate variability.

### Important Drought and Heat Tolerance Traits

Grain components between treatments were positively but weakly correlated, indicating that accessions which performed well under D, were often susceptible to the combination of both drought and heat stresses. Accessions which performed well under both stresses were mostly Australian and Mexican varieties, which have been selected for their yield performance in dry and hot climates and represented about 70% of the diversity panels. However, approximately one fifth of the tolerant accessions were varieties from various origins such as the Middle East, Central Africa, the United States, Canada, and India. Landraces from Middle Eastern countries, which represented only about 7% of the panels, accounted for approximately 6% of the tolerant accessions in 2017. Of the number of accessions represented in both diversity panels, all three types (i.e., landraces, varieties from Australia and Mexico, and varieties from other origins) accounted for approximately one third of the tolerant accessions.

Grain number was mostly increased by the same allele as grain weight in both GWAS and NILs, indicating an important factor under post-anthesis drought and heat stress. Grain number is known to be affected by pre-anthesis stress (Fabian et al., 2019) but has also been found to be decreased by post-anthesis stress (Prasad et al., 2011; Qaseem et al., 2018). Grain number, in our experiments, accounted for only well-filled grains (i.e., grains of size > 2.0 mm). The trait therefore represents grain filling ability. The allele increasing grain number also promoted single grain weight and thousand kernel weight with a significant increase in thousand kernel weight in NIL pairs 3 and 4. In NILs, spike length, spike number, as well as screenings (i.e., percentage of small, empty or partly filled grains) were negatively associated with grain weight under DH and were increased by the opposite allele than grain weight. A reduced tiller and initial grain set (sink strength) might be therefore an advantage when followed by combined drought and heat stress during grain-filling due limited assimilate availability (source strength) (Gupta et al., 2011).

Leaf water potential was the physiological trait with the strongest correlation with grain weight. Plants which maintained a less negative leaf water potential during stress had an increased grain weight, suggesting the role of this trait as both a stress and a stress tolerance indicator. Plants with a less negative leaf water potential had also a higher harvest index and a reduced spike number. Apart for the potential advantage of a limited sink strength, the reduced spike number and thus reduced surface area might have led to a decrease in transpiration rate (i.e., water loss) and water use in comparison to plants with more spikes. This would be especially important under severe drought and heat stress conditions (Tricker et al., 2018).

### Phenology and Plant Height Independent QTL

Significant marker-trait associations (MTA) were initially selected using the Bonferroni threshold (i.e., -log10 (p) ≥ 5.68), however, we could not find any marker associated with grain weight or grain weight components under D or DH. Due to the high stringency of the Bonferroni threshold, type II error (i.e., false negative) is inflated drastically reducing the power of detection of loci with smaller allelic effects especially of more complex traits such as yield. In contrast, a low threshold bears the risk of increasing the detection of false-positive MTA (type I error) (Hamblin et al., 2011). We, therefore, chose FDR of 0.05 and 0.20 which have been considered sensible measures to balance between the type I error and type II error in GWAS (Benjamini and Hochberg, 1995; Storey and Tibshirani, 2003). To minimize the risk of potential false positive markers, we only considered QTL which co-located with at least one other QTL. Using the FDR as thresholds, we found and validated a strong QTL on chromosome 6A, confirming the findings from our GWAS.

Some of the QTL identified here were associated with well-known genes that are commonly used in marker assisted selection. For instance, days to anthesis was associated with the photoperiod sensitive gene *Ppd-D1* on chromosome 2D. Even though *Ppd-D1* has been shown to affect grain yield (Arjona et al., 2018; Mason et al., 2018), no significant association between *Ppd-D1* and grain weight or its components was found in this study, regardless whether grain traits were adjusted or not for days to anthesis. Eight additional QTL for days to anthesis were identified on chromosomes 2A, 4A, 5A, 5D, and 7B. The QTL on the short arm of chromosome 2A (*QDTA.aco-2A.1*) and the long arm of chromosome 5A (*QDTA.aco-5A.2*) were located in close proximity to the photoperiod sensitive gene *Ppd-A1* and the vernalization gene *Vrn-A1*, respectively (Yan et al., 2004; Wilhelm et al., 2009). The second QTL on chromosome 5A (*QDTA.aco-5A.1*) co-located with a QTL for plant height (*QPH.adh-5A*, non-adjusted and adjusted for anthesis) and QTL for days to heading and anthesis under combined drought and heat field conditions identified by Maccaferri et al. (2008) and Pinto et al. (2010). QTL for days to maturity under well-watered conditions and plant height were reported (Zanke et al., 2014; Qaseem et al., 2019) close to the ones identified in this study for days to anthesis and plant height on chromosome 5D (*QDTA.aco-5D*, *QPH.adh-5D*—non-adjusted and adjusted for anthesis). The QTL for plant height on chromosome 2B (*QPH.adh-2B*) co-located with the one previously detected by Sun et al. (2017).

Overall, 134 out of 145 identified QTL for grain weight were not related to days to anthesis or plant height. A pot-based system enabling the individual treatment of plants seemed therefore to be advantageous for the identification of QTL associated with drought and heat stress tolerance in comparison to field trials. However, drought and heat tolerance traits influenced by pot size such as root architecture, biomass, and spike number might need to be analyzed in a different setting as low correlations between these traits and grain weight in our GWAS indicated.

### Novel QTL for Drought and Heat Tolerance

Allelic effects for grain weight and grain weight components were low to moderate ranging between 3.3% and 21.2% as often the case for complex traits such as yield (Hamblin et al., 2011; Zanke et al., 2015). Nevertheless, several major QTL for grain weight and its components were identified in this study. Identified QTL for grain weight co-located with previously detected QTL in wheat, except for *QGWt.ara-6A.3*. While half of the previously identified QTL have been associated with yield components and a third were controlling yield or grain weight itself, only four have previously been identified under combined drought and heat stress.

Important loci associated with grain weight under drought, heat or the heat response under drought were identified on chromosomes 1A, 2D, 3A, 3B, 3D, 5B, 6B, 6D, 7A, and 7B, of which most grain weight-related QTL were located on 7B. Apart from grain weight, the loci were pleotropic for harvest index, screenings, grain number, single grain weight, and leaf water potential. Ten of the 16 identified QTL for grain weight corresponded to QTL identified by Sun et al. (2017), of which two, located on chromosomes 1A and 7A, regulated grain weight per spike. Six other QTL associated with grain weight were detected under well-watered, rainfed, or heat conditions (Sukumaran et al., 2015; Valluru et al., 2017; Wang et al., 2017; Qaseem et al., 2018) and coincided with the QTL for grain weight under DH and the heat response under drought on chromosome 3B, 5B, and the long arm of chromosome 6B (*QGWp.adh-3B.1*, *QGWp.adh-3B.2*, *QGWp.adh-5B*, *QGWt.ara-6B.7*, *QGWp.ara-6B.3*). The QTL on the long arm of chromosome 6B also co-located with QTL for harvest index under combined drought and heat stress (Garcia et al., 2019) as well as single grain weight and leaf chlorophyll content under heat (Shirdelmoghanloo et al., 2016). Regions on chromosome 3B co-located with QTL for tiller number under combined drought and heat stress, grain number, biomass, and harvest index under well-watered conditions (Qaseem et al., 2018), while the region on chromosome 7A coincided with QTL for spike length and water-soluble carbohydrates (Gao et al., 2015; Dong et al., 2016; Sun et al., 2017).

Seven of the here identified genomic regions which have not been previously associated with grain weight itself were located on chromosomes 2D, 3A, 3D, 6B (short arm), 6D, and 7B. QTL previously found on chromosomes 2D, 3A, 3D, 6D, and 7B for grain number (Gao et al., 2015; Shi et al., 2017; Sun et al., 2017), on chromosomes 3A, 3D, and 6D for thousand kernel weight (Zanke et al., 2015; Sun et al., 2017), and on chromosomes 1A, 3A, 3D, 6B, and 6D for grain size (Sun et al., 2017) were mapped to similar positions in the wheat reference genome sequence (RefSeq v1.0) to the QTL identified in this study. Under controlled heat conditions, Shirdelmoghanloo et al. (2016) identified QTL for grain filling duration, flag leaf length, shoot length, and harvest index which coincided with QTL for grain weight and harvest index for the heat response under drought on chromosome 7B (*QGWt.ara-7B.1*, *QGWp.ara-7B*, *QHI.ara-7B.1*). QTL associated with leaf chlorophyll content co-located with QTL on chromosome 2D (Gao et al., 2015).

### Stable QTL for Yield and Yield Components

Seven genomic regions on chromosomes 3B, 4A, 5B, 6A, 6B, and 7B were particularly of interest as they were stable across treatments, traits, and years. Three of the genomic regions, located on chromosome 4A, 6B, and 7B, were associated with grain weight for the heat response under drought, harvest index, biomass, screenings, and leaf water potential. QTL for grain weight under single and combined drought and heat stress co-located with the QTL on chromosomes 6B and 7B (Valluru et al., 2017; Garcia et al., 2019; Qaseem et al., 2019) and QTL for harvest index and biomass were previously found under heat conditions on chromosome 7B (Qaseem et al., 2019). Using two QTL were reported, mainly wheat accessions from China, for grain number at all three regions (Shi et al., 2017; Sun et al., 2017). Further QTL for water-soluble carbohydrates, normalized difference vegetation index, and canopy temperature depression were found in 3–10 Mbp distance on RefSeq v1.0 on chromosomes 4A and 6B (Sukumaran et al., 2015; Dong et al., 2016; Gao et al., 2016). The genomic region on chromosome 5B regulated a total of four traits including grain weight per primary tiller, leaf water potential, and single grain weight per primary tiller and per plant. Sun et al. (2017) and Wang et al. (2017) reported QTL for thousand kernel weight and spike number at this region. The QTL on 3B co-located with QTL for anther extrusion (Muqaddasi et al., 2017).

On the short arm of chromosome 6A, we detected QTL for the heat response under drought of grain weight per primary tiller and per plant, grain number per plant, single grain weight per plant and harvest index, as well as screenings per primary tiller and per plant under D and DH (*QGWt.ara-6A.1*, *QGWp.ara-6A*, *QGNp.ara-6A*, *QSGWp.ara-6A*, *QHI.ara-6A.1*, *QSCRt.adh-6A*, *QSCRp.adr-6A.1*). QTL for grain weight, grain number, spike length, and tiller number in proximity to our region were associated with well-watered and drought conditions, but not with combined drought and heat stress (Sun et al., 2017; Qaseem et al., 2018; Qaseem et al., 2019). In fact, no QTL for grain weight under combined drought and heat stress has been identified at this locus to date, making it thus a promising target for the discovery of novel genes under drought and heat stress.

### Field Validation

We developed NILs which differed at the 6A target QTL to validate our findings from the GWAS. By using an existing nested association mapping population, we were able to rapidly introduce the allele commonly distributed in Asian and African landraces into an Australian elite cultivar background. Findings were in accordance with the results from the GWAS in 2016, except for NIL pair 2. Results of the NIL pair 2 showed an opposite but not significant trend from the other three NIL pairs for most of the grain traits per plot (i.e., spike number, biomass, screenings, grain number, grain weight, and harvest index). Even if the genotyping results assumed uniformity among all four NIL pairs at the target region, the developed markers might not cover the target region sufficiently and differences in recombination events might not be visible. The genetic background might also be different between different pairs, containing potential cis- or trans- regulating elements controlling the target region. A whole genome sequencing of all NILs might therefore be required.

Clear trends were visible for all measured traits and significant differences were observed for six of the eight grain related traits. To potentially increase the statistical power by an increased sample size, traits of five randomly chosen spikes for each plot were measured. High correlation between measurements made per plot and per single spikes were observed for screenings and single grain weight, whereas grain number and grain weight per spike were not representative of the entire plot explaining the inconsistency of grain number per spike in comparison to the rest of the results. Even though grain weight per spike and per plot were not significantly correlated, both were increased in the NILs carrying the allele donated by the exotic parent in comparison to NILs carrying the allele donated by the adapted parent among three of the four NIL pairs. The biggest increase of grain weight was found in NIL pair 3 with 26.4% which would mean an immense yield gain in dry and hot environments. In both GWAS and the QTL validation, we applied severe DH stress, probably causing an inflation of the effect of the allele. We therefore would not expect an impact of 26.4% under actual field conditions but the incorporation of this allele could still be a significant contribution to future wheat breeding.

The GWAS and the validation of the QTL also showed an independency between this locus and QTL from plant phenology. This is important considering most studies in field conditions show a strong effect of *Ppd-D1* that can potentially mask other loci affecting grain weight (Mason et al., 2018; Garcia et al., 2019). The use of a semi-controlled pot system allowed us to treat plants individually and to identify several QTL for D, DH, and heat response under drought. To confirm the effect of our target QTL in actual field conditions, the testing of NIL pairs in multi-environment trials over several years is required. A semi- or completely controlled pot system might therefore be a useful and cost-effective approach for the preliminary detection of QTL.

## Data Availability Statement

The raw data supporting the conclusions of this article will be made available by the authors, without undue reservation, to any qualified researcher.

## Author Contributions

PT and DF conceived the study. JS, PT, PE, MG, and DF designed the experiments. JS and PK conducted the experiments. JS wrote the manuscript and performed the statistical analysis. PE gave support in the statistical analysis. PT, PE, PK, MG, and DF edited the manuscript.

## Funding

The project was funded by the Australian Research Council Industrial Transformation Research Hub for Genetic Diversity and Molecular Breeding for Wheat in a Hot and Dry Climate (project number IH130200027).

## Conflict of Interest

The authors declare that the research was conducted in the absence of any commercial or financial relationships that could be construed as a potential conflict of interest.
