# Supplementary material for: Novel Alleles for Combined Drought and Heat Stress Tolerance in Wheat
Source: Front Plant Sci. 2020 Jan 31;10:1800. doi: 10.3389/fpls.2019.01800 (PMC7005056; doi:10.3389/fpls.2019.01800)
Supplement: Supplementary file 1 [file Table_1.docx]

## Supplementary Figures

**Supplementary Figure 1.** Split-plot design in 2016 and 2017. Border plants are marked in blue, plants which were exposed to drought stress in yellow and plants exposed to combined drought and heat in red. Plants were distributed over four tables and randomized over three blocks (i.e. one replicate per block) with each block containing 18 columns and 35 rows of pots. Pots were randomized differently in each year to avoid that accessions were located at the same spot as the year before.

**A**


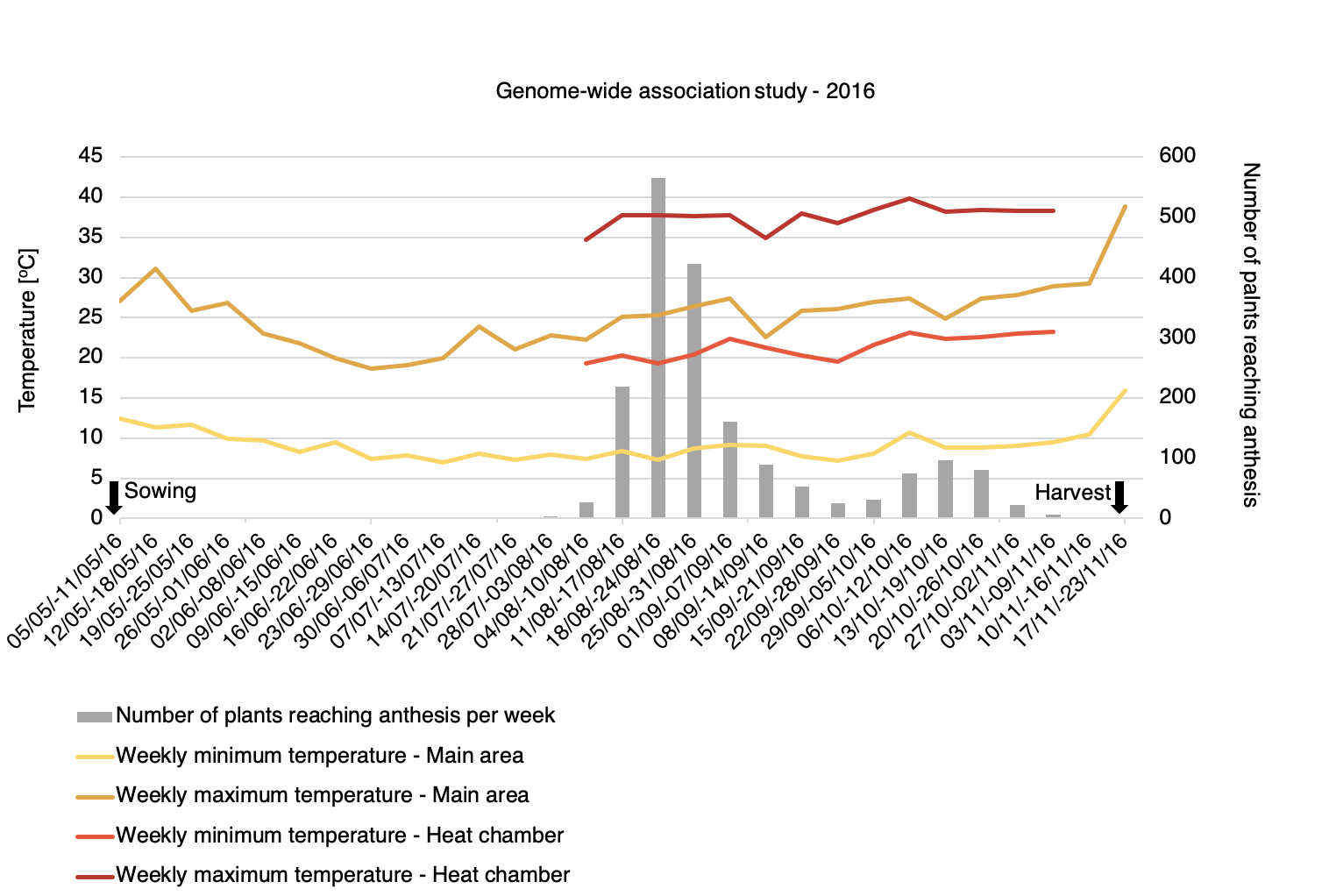


**B**

**
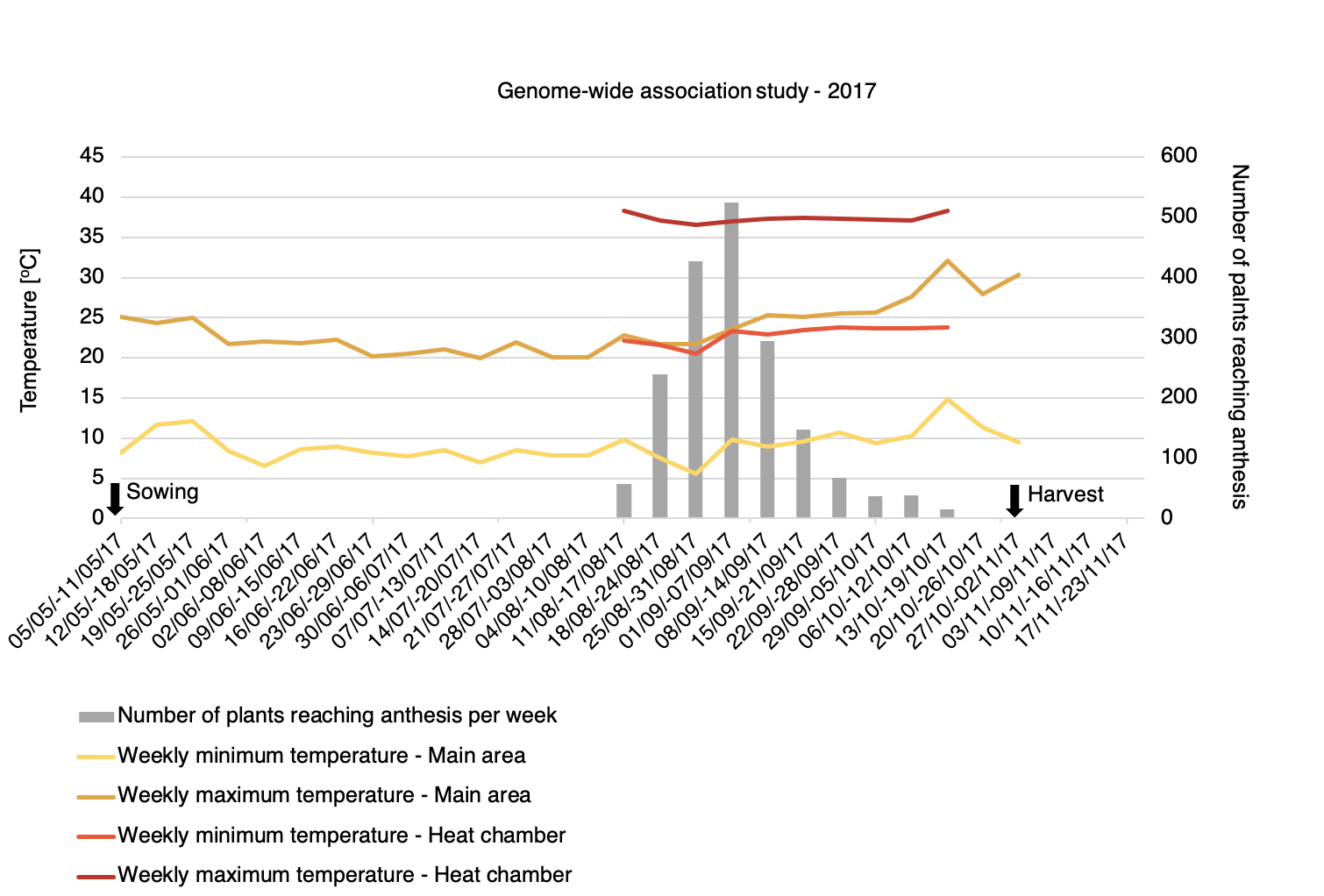
**

**C**

**
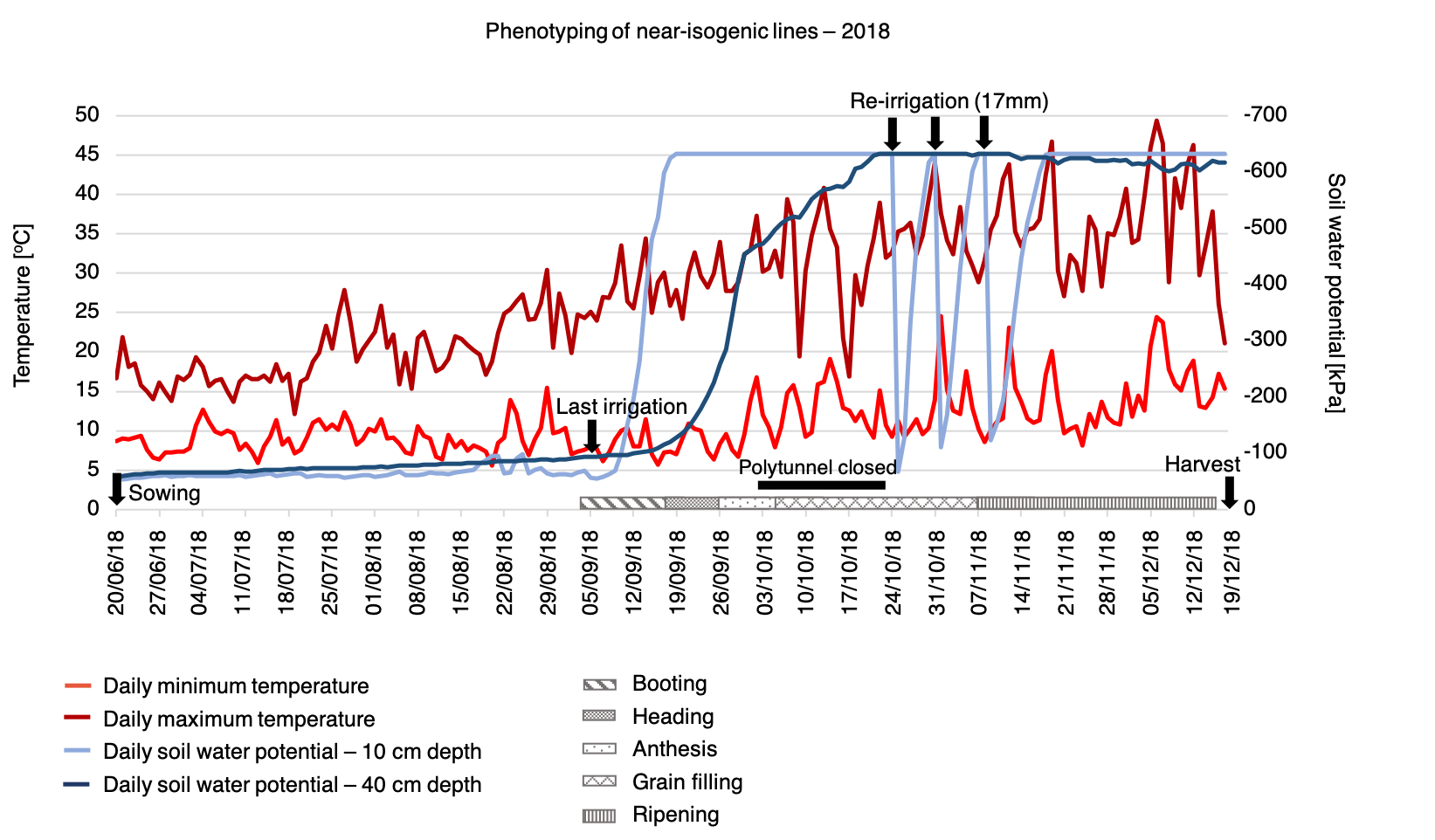
**

**Supplementary Figure 2**. Experimental conditions in (A) 2016, (B) 2017 and (C) 2018. Graphs of 2016 and 2017 experiments include sowing and harvest date, minimum and maximum temperatures and number of plants reaching anthesis per day. The graph of the 2018 experiment includes sowing and harvest date, minimum and maximum temperatures, soil water potential and phenology stage.

Grain number plant ^-1^

Grain number primary tiller ^-1^

Single grain weight plant ^-1^ (mg)

Grain weight plant ^-1^ (g)

Grain weight primary tiller ^-1^ (g)

Single grain weight primary tiller ^-1^ (mg)

**Supplementary Figure 3.** Boxplots of phenotypic traits measured in 2016 and 2017 under drought (yellow) and combined drought and heat stress (red). For days to anthesis, values of D and DH treated plants were combined (black). Different superscript letters indicate significant differences (p ≤ 0.05) based on Tukey’s HSD test.

**A**

Grain weight plant ^-1^ – Drought 2017

Grain weight plant ^-1^ – Drought and heat 2017

**C**

Grain weight plant ^-1^ – Ratio 2017

**Supplementary Figure 4.** Manhattan plots and Q-Q plots of grain weight per plant under (A) drought, (B) combined drought and heat and (C) the ratio (i.e. heat response under drought) in 2017. Dark blue lines within Manhattan plots correspond to a threshold of a false discovery rate of 0.05, bright blue lines to a of a false discovery rate of 0.20. Green dots correspond to significant markers trait associations. 1A - 7D, wheat chromosomes; U, unlinked markers.
